# Improving attitudes towards the COVID-19 vaccine in forensic mental health workers: a quality improvement project

**DOI:** 10.1192/bjo.2021.523

**Published:** 2021-06-18

**Authors:** Harrison Howarth, Toby Stevens, Benjamin Gostick, Lisanne Stock, David Stone

**Affiliations:** North London Forensic Service

## Abstract

**Aims:**

The primary aim of the project was to improve attitudes towards the COVID-19 vaccine in forensic mental health staff at a large regional tertiary forensic psychiatry unit. The main variable examined was attitudes towards safety of the vaccine. Secondary aims included decreasing misinformation about the vaccine and improving vaccine uptake.

**Method:**

Paper questionnaires were distributed to willing staff members across 6 forensic inpatient wards within the North London Forensic Service. Participants included a range of allied health professionals including nurses, health care assistants, ward managers, occupational therapists, assistant therapists and administrative staff. Questionnaires used a mixture of Likert scale for agreement/disagreement with statements and yes/no questions.

Plan-Do-Study-Act (PDSA) methodology was utilised in implementing changes, and repeat questionnaires used to measure changes in attitude and behaviour. Change ideas implemented included the creation of ‘mythbusters’ posters which target vaccine misinformation, the creation and distribution of posters of staff members who had already taken their vaccine, the creation of vaccine champions to aid engagement in conversation about the vaccine, vaccine information packs being distributed to all wards and the opportunity for staff to ‘drop-in’ to clinics for information about the vaccine.

**Result:**

Vaccine uptake improved from 7% before interventions to 69% after interventions.

The proportion of people very unlikely or unlikely to get the vaccine reduced from 25% to just 9%. The proportion of those feeling neutral reduced from 32% to 6%. The proportion of those either likely or very likely to get the vaccine increased from 34% to 85%.

Before interventions only 20% felt that the vaccine was either safe or very safe. This improved to 63% after interventions

Before interventions, only 27% of respondents felt they had received enough information by the trust to make an informed decision. After interventions, 80% said they had received enough information.

The project was successful in reducing misinformation in every domain. Particularly reassuring was the reduction to zero of some of the most harmful misinformation claims, such as the presence of a tracking chip in the vaccine and the belief that COVID does not exist.

71% of respondents indicated the interventions we set out changed their view on the COVID-19 vaccine.

**Conclusion:**

The changes implemented lead to clear improvements in all domains measured, suggesting targeted information is an effective strategy in improving uptake and attitudes around the vaccination program.

